# Traditional Chinese Medicine Enhances Survival in Patients with Gastric Cancer after Surgery and Adjuvant Chemotherapy in Taiwan: A Nationwide Matched Cohort Study

**DOI:** 10.1155/2021/7584631

**Published:** 2021-02-10

**Authors:** Wei-Tai Shih, Pei-Rung Yang, Yi-Chia Shen, Yao-Hsu Yang, Ching-Yuan Wu

**Affiliations:** ^1^Department of Traditional Chinese Medicine, Chang Gung Memorial Hospital, Chiayi, Taiwan; ^2^School of Traditional Chinese Medicine, College of Medicine, Chang Gung University, Tao-Yuan, Taiwan; ^3^Health Information and Epidemiology Laboratory, Chang Gung Memorial Hospital, Chiayi, Taiwan

## Abstract

**Background:**

Gastric cancer remains a major cancer globally. More than half of patients with gastric cancer undergo surgery in Taiwan; however, few large nationwide studies have investigated the effects of traditional Chinese medicine (TCM) on gastric cancer management after surgery. This study aimed to evaluate the effect of TCM on patients with gastric cancer following surgery and adjuvant chemotherapy in Taiwan and its prescription trends.

**Methods and Materials:**

The cohort sampling data set was obtained from the Registry of Catastrophic Illness Patient Database, a research database of patients with severe illnesses from the National Health Insurance Research Database, Taiwan. Patients who had received a new diagnosis of gastric cancer and had undergone surgery were enrolled. We matched TCM users and nonusers at a ratio of 1 : 3 based on the propensity score, and TCM users were also grouped into short-term and long-term users.

**Results:**

The number of TCM users and nonusers was 1701 and 5103 after applying the propensity score at a ratio of 1 : 3. Short-term users and long-term TCM users were independently associated with a decreased risk of death with HRs of 0.59 (95% confidence interval (CI), 0.55–0.65) and 0.41 (95% CI, 0.36–0.47), respectively, compared with TCM nonusers. We also obtained similar results when we adjusted for covariates in the main model, as well as each of the additional listed covariates. We also observed similar HR trends in short-term users and long-term TCM users among men and women aged <65 years and ≥65 years. The most commonly prescribed single herb and herbal formula in our cohort were Hwang-Chyi (Radix Hedysari; 11.8%) and Xiang-Sha-Liu-Jun-Zi-Tang (15.5%), respectively.

**Conclusion:**

TCM use was associated with higher survival in patients with gastric cancer after surgery and adjuvant chemotherapy. TCM could be used as a complementary and alternative therapy in patients with gastric cancer after surgery and adjuvant chemotherapy.

## 1. Introduction

Gastric cancer, including adenocarcinomas and other carcinomas, remains a major cancer globally and is ranked fifth among the most frequently diagnosed cancers, with over 1, 000, 000 new cases reported in 2018, and it is the third leading cause of cancer-related death, with approximately 7, 83, 000 deaths. [1] The incidence rate of gastric cancer is markedly high in Eastern Asia with 32.1 per 1, 00, 000 among men and 13.2 among women, and the incidence rate in South Korea is approximately 60 per 1, 00, 000 new cases annually among men and 25 per 1, 00, 000 among women. [2] Surgical resection is often adopted in the early stages of gastric cancer, whereas adjuvant therapies, such as radiotherapy or chemotherapy, are often considered in the advanced stages of gastric cancer. [3, 4] In 2010–2014, the five-year survival rate of gastric cancer was 69% in South Korea, 60% in Japan, and less than 40% in most other countries, including Taiwan and the United States. [5].

Traditional Chinese medicine (TCM), traumatology manipulative therapies, and acupuncture, which are included in traditional Chinese medical in Taiwan, are widely accepted as some of the most popular complementary and alternative (CAM) therapies for patients with cancer. In addition, Chinese herbal products (CHPs) represent decoctions that have been adopted more recently based on their consistent quality and are applied in clinical settings. In addition, because of their quality, convenience, and approval for full reimbursement for both single herbs and herbal formulas CHPs by the National Health Insurance (NHI) in Taiwan, Chinese medical physicians prescribe them to their patients. A computerized reimbursement database of the NHI, the National Health Insurance Research Database (NHIRD), contains comprehensive data on both TCM and Western medicine. Consequently, the NHIRD offers an appropriate resource for conducting pharmacoepidemiological research on medicine use.

A previous study reported that TCM improved overall survival among patients with gastric cancer in Taiwan. [6] In Taiwan, most patients with gastric cancer undergo surgery, and more than half of the patients who undergo surgery receive chemotherapy afterward. There are many clinical studies on the treatment of gastric cancer by TCM after surgery and chemoradiotherapy in China. However, large nationwide studies on the potential effects of the use of TCM on patients with gastric cancer after surgery and adjuvant chemotherapy remain limited. Therefore, the aim of the present study was to evaluate the effect of the use of TCM on patients with gastric cancer following surgery and adjuvant chemotherapy in Taiwan and the associated TCM prescription trends.

## 2. Methods

### 2.1. Data Source

The cohort sampling data set of the present study was obtained from the Registry of Catastrophic Illness Patient Database (RCIPD), a research database of patients with severe illnesses within the NHIRD system, NHI, Taiwan.

Because the Taiwanese NHI is obligatory for all residents, the NHIRD, which covers almost the entire population of 23.7 million people, is a rather detailed database in a health-related field.

Using data from the RCIPD, we can gather information based on both admissions and outpatient visits, which contains patient data such as admission and discharge dates, sex, age, diagnoses (made according to the International Classification of Diseases, Ninth Revision, Clinical Modification [ICD-9-CM] codes), and outpatient visits. In addition, data include prescription for patients, such as the prescribed medicine, initiation date, duration, dosage, and total expenditure.

Because the information in the database is anonymous, in line with personal electronic data protection regulations set by the National Health Research Institutes of Taiwan, NHIRD data can only be used for research purposes. The use of the database, in which information is anonymized and informed consent requirement is waived, was approved by the Ethics Review Board of Chang Gung Memorial Hospital, Chiayi Branch, Taiwan.

### 2.2. Study Subjects

The study cohort was selected from admission and outpatient department records using diagnostic variables in the RCIPD. The ICD-9-CM codes 151 and codes beginning with 151 were used for the identification of gastric cancer. We included patients who had received a diagnosis of gastric cancer between January 1, 1999, and December 31, 2008, and survival was tracked until December 31, 2013. The endpoint of observation was death or end of follow-up. We excluded patients who had previously received a diagnosis of other cancer. In addition, we excluded patients who had not undergone surgery, patients who had undergone chemotherapy before surgery, and patients who did not undergo chemotherapy after surgery. In the consequence, patients with gastric cancer after surgery and adjuvant chemotherapy were included, whether they have done radiotherapy or not. Patients with missing information related to age, sex, level of urbanization, or level of income were also excluded.

### 2.3. Traditional Chinese Medicine Exposure

We downloaded data on reimbursements for Chinese medicine from the NHI website. We obtained the corresponding drug data from the Committee on Chinese Medicine and Pharmacy website, including the proportions of each constituent, date and period of drug approval, drug names, and manufacturers' codes.

To classify patients into TCM users and nonusers, patients who used TCM for 30 days or more were considered TCM users and those who used TCM for less than 30 days were considered TCM nonusers. In addition, to enable the evaluation of a dose-response relationship, we further grouped TCM users into short-term TCM users (patients who used TCM for 30–179 days) and long-term TCM users (patients who used TCM for more than 180 days).

### 2.4. Potential Confounders

We identified some comorbidities as potential confounding risk factors for gastric cancer, including the following diagnoses recorded during the study period: diabetes mellitus (ICD-9-CM codes 249-250), hypertension (ICD-9-CM codes 401-405), alcoholism (ICD-9-CM code 303), smoking-related disorder (ICD-9-CM code 305.1), chronic renal failure (ICD-9-CM code 585), and liver cirrhosis (ICD-9-CM codes 571.2, 571.5, and 571.6). We also considered age, sex, monthly insurance income, and urbanization level in the model. Chemotherapy treatments were classified into epirubicin- and mitomycin-based treatments, taxanes, and other regimens.

### 2.5. Matched Cohort

By using propensity scores, TCM users and nonusers were perfectly matched at a ratio of 1 : 3, and it is a great estimate of how likely a patient would use TCM as a treatment. Background information, such as urbanization level, monthly insurance income, age, sex, and all comorbidities, was obtained.

### 2.6. Statistical Analysis

We compared the distribution of baseline characteristics, namely sex, age at surgery, urbanization level, income level, comorbidities, and chemotherapy regimen, between TCM users and nonusers.

We used the Kaplan–Meier method to estimate the cumulative probability of the overall survival of TCM users and nonusers. We performed the log-rank test to examine differences in overall survival between the cohorts. We used the Cox proportional hazards model to compute hazard ratios (HRs) at a 95% confidence interval (CI) after adjustment for age, sex, monthly insurance income, urbanization level, and chemotherapy regimen. A two-tailed *P* value of 0.05 was considered significant. All analyses were conducted using SAS v9.4 (SAS Institute, Cary, NC, USA).

## 3. Results

**Figure 1:**
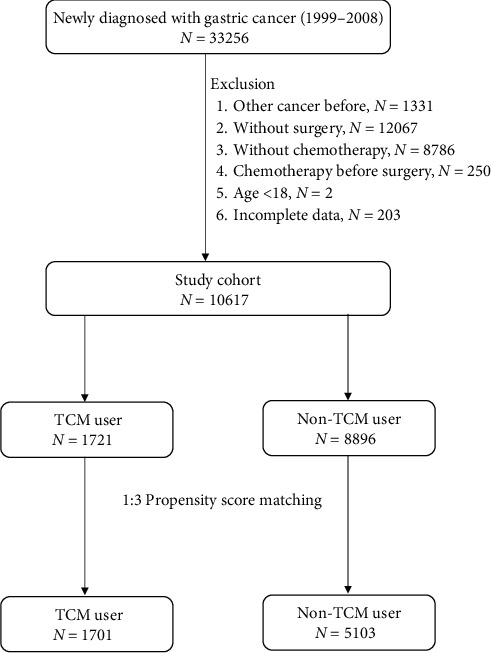
Flow chart of the patient enrollment process for the study cohort and the matched cohort.

Of 33, 256 patients who had received a new diagnosis of gastric cancer between 1999 and 2008, 1331 were excluded because they had received a prior diagnosis of another cancer, 21 103 were excluded because they did not undergo surgery, chemotherapy, or surgery after chemotherapy, 2 were excluded because they were aged less than 18 years, and 203 were excluded because their data were incomplete. A total of 1721 and 8896 patients were classified as TCM users and nonusers, respectively. After applying the propensity score at a ratio of 1 : 3, the number of TCM users and nonusers was 1701 and 5103, respectively (Figure 1).

In our study cohort, the mean age at surgery of TCM users and nonusers was 59.8 and 59.7 years, respectively. No significant differences were noted between the percentage of TCM users and nonusers based on sex, age at surgery, urbanization level, income level, and comorbidities (Table 1).

**Figure 2:**
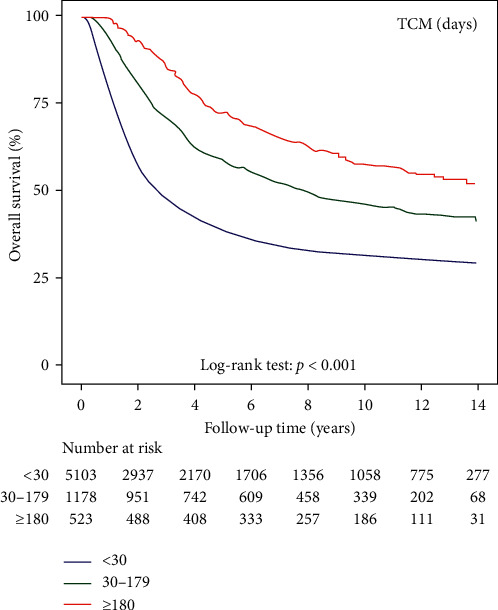
Kaplan–Meier curves of overall survival in patients with gastric cancer after surgery and adjuvant chemotherapy among TCM nonusers, short-term TCM users, and long-term TCM users.

Table 2 shows overall survival in patients with gastric cancer among TCM nonusers, short-term TCM users, and long-term TCM users.  Figure 2 shows Kaplan–Meier curves for overall survival in patients with gastric cancer among TCM nonusers, short-term TCM users, and long-term TCM users.

After adjusting for sex, age, urbanization level, and income level in the main model, short-term and long-term TCM users were independently associated with a decreased risk of death with HRs of 0.59 (95% CI, 0.55–0.65) in TCM users and 0.41 (95% CI, 0.36–0.47) in TCM nonusers. We obtained similar results when covariates were adjusted for in the main model, as well as for each additional covariate, namely, diabetes mellitus, hypertension, alcoholism, smoking-related disorder, chronic renal failure, liver cirrhosis, and chemotherapy regimen. In the subgroup analysis of sex and age, we observed similar HR trends in short-term and long-term TCM users among men (0.62 [95% CI, 0.55–0.69] and 0.45 [95% CI, 0.38–0.54], respectively), women (0.56 [95% CI, 0.49–0.64] and 0.36 [95% CI, 0.29–0.45], respectively), those aged <65 years (0.60 [95% CI, 0.54–0.67] and 0.38 [95% CI, 0.31–0.45], respectively), and those aged ≥65 years (0.58 [95% CI, 0.51–0.66] and 0.46 [95% CI, 0.38-057], respectively) (Table 3).

Table 4 lists single herbs and herbal formulas most commonly prescribed for gastric cancer after surgery and adjuvant chemotherapy in our cohort, including the prescription frequencies, average prescription durations, and average daily doses. The single herb most commonly prescribed in our cohort was Hwang-Chyi (Radix Hedysari; 11.8%), followed by Dan-Shen (Radix Salviae Miltiorrhizae; 9.8%), Yan-Hu-Suo (Rhizoma Corydalis; 9.4%), Bai-Hua-She-She-Cao (Herba Hedyotidis Diffusae; 9.2%), and Hou-Pu (Cortex Magnoliae; 9.0%). Xiang-Sha-Liu-Jun-Zi-Tang (15.5%) was the herbal formula most commonly prescribed in our cohort, followed by Ping-Wei-San (12.6%), Ban-Xia-Xie-Xin-Tang (11.8%), Bu-Zhong-Yi-Qi-Tang (10.3%), and Shen-Lin-Bai-Zhu-San (10.2%).

The daily doses of the most commonly prescribed single herbs and herbal formulas were in the ranges of 1.4–1.7 g and 4.0–4.9 g, respectively. The average duration of single herbs and herbal formulas most commonly prescribed ranged from 7.8 to 11.1 days.

## 4. Discussion

This study has several merits. Because our population data set was directly obtained from the RCIPD, our study enrolled all patients with gastric cancer between 1999 and 2008 in Taiwan instead of a representative sample. In addition, our study cohort was obtained from the RCIPD, which is highly accurate due to its high demand for implicating catastrophic illness.

A previous study demonstrated that TCM enhanced the survival of patients with gastric cancer. [6] As the tumor stage could not be defined, the previous study could not reveal the potential relationship between adopted therapies and differences in disease severity. However, because in the present study we examined patients with gastric cancer who underwent chemotherapy following surgery, our study cohort had similar disease severity levels and stages. Based on propensity score matching, we increased homogeneity between TCM users and nonusers and reduced the number of nonmatched users as much as possible. After propensity score matching, only 20 (1.16%) of 1721 TCM users could not be matched in our study, whereas more than 25% of TCM users could not be matched in the previous study. In addition, we classified TCM users into short-term users who used TCM for less than 180 days and long-term users who used TCM for 180 days or more to observe the effect of long-term TCM use. To verify our results, we adjusted additional covariates, namely, diabetes mellitus, hypertension, alcoholism, smoking-related disorder, chronic renal failure, liver cirrhosis, and chemotherapy regimen, to examine whether results were consistent, and similar results in subgroup effects based on sex and age at surgery also confirmed our findings. Finally, because survival was calculated from the day TCM was taken in our study, we reduced immortal time bias as much as possible.

In Taiwan, CHPs are considered a modern form of decoctions because of their more consistent quality when compared with TCM. The NHI program reimburses patients fully for both single-herb products and multi-herb products prescribed. Chinese medical physicians used to prescribe one or more herbal formulas in combination with several single herbs in each prescription according to the state of illness of a patient.

In our study, half of the single herbs and herbal formulas most commonly prescribed that are listed in Table 4 are often used to treat ailments of the gastrointestinal system. Xiang-Sha-Liu-Jun-Zi-Tang, the most commonly prescribed herbal formula identified in the present study, was often used to treat upper gastrointestinal disorders, including indigestion, gastroesophageal reflux, and anorexia, [7, 8] and has been reported to prevent nausea, vomiting, and anorexia induced by chemotherapy in patients with cancer [9–11] and exert beneficial effects on gastrointestinal disorders, anorexia, and postoperative gastric ileus when used in combination with Western drugs. [12, 13] Ping-Wei-San is also often prescribed for the treatment of upper gastrointestinal disorders, including esophageal reflux, gastritis, gastric or duodenal ulcers, and enteritis. [14–16] Furthermore, Ban-Xia-Xie-Xin-Tang has been reported to reduce diarrhea during chemotherapy [17, 18] and could prevent chemotherapy-induced oral mucositis in esophageal cancer [19] and in chemoradiation of head and neck cancers. [20] Shen-Ling-Bai-Zhu-San is one of the most common formulas used for the treatment of ulcerative colitis [21, 22] and has also been reported to reduce gastric cancer chemotherapy-induced toxicity. [23] Hou-Pu (Cortex Magnoliae) had been demonstrated to be beneficial for the treatment of gastrointestinal disorders. [24] Huang-Chyi (Radix Hedysari), which was the most commonly prescribed single herb in our study, and Bu-Zhong-Yi-Qi-Tang have been demonstrated to alleviate cancer-related fatigue. [25–27] Dan-Shen (Radix Salviae Miltiorrhizae) and Bai-Hua-She-She-Cao (Herba Hedyotidis Diffusae) have been shown to have anticancer effects, [28–30] whereas Bai-Hua-She-She-Cao (Herba Hedyotidis Diffusae) is the most common single herb prescribed for patients with colon cancer and breast cancer in Taiwan. [31, 32] Finally, Yan-Hu-Suo (Rhizoma Corydalis) showed pain control benefits. [33, 34].

Our study has some limitations that should be addressed. First, the NHI program only reimburses the cost of CHPs prescribed by Chinese medical physicians. Therefore, CHPs or decoctions purchased directly from TCM pharmacies were not included in our analysis. Consequently, the frequency of TCM use could have been underestimated in the present study. Nevertheless, because the NHI program has a comprehensive cover for CHPs prescribed by Chinese medical physicians, which is generally lower than the cost of CHPs or decoctions sold in TCM pharmacies, the underestimation would likely be minimal. In the present study, we could not verify exact dosages taken by enrolled patients. We could only assume that patients took medications as prescribed, which may overestimate the actual ingested dosage because some degree of noncompliance is always anticipated. Furthermore, detailed information, such as living environment, lifestyle, nutrition, and other examination data, were not available from the RCIPD. Finally, the stages of cancer were not recorded in the RCIPD. Nevertheless, we controlled the severity of cancer in our study cohort to some extent by selecting patients with gastric cancer undergoing chemotherapy after surgery.

## 5. Conclusions

In conclusion, in our study, TCM use was associated with higher survival in patients with gastric cancer after surgery and adjuvant chemotherapy. TCM could be adopted as a CAM therapy in patients with gastric cancer after surgery and adjuvant chemotherapy.

## Figures and Tables

**Table 1:** Baseline patient characteristics of TCM users and nonusers.

Variables	TCM user (*N* = 1701)		Non-TCM user (*N* = 5103)		*P* value^1^
	*n*	%	*n*	%	
Sex					0.2554
Female	722	42.5	2086	40.9	
Male	979	57.6	3017	59.1	
Age at surgery, years					0.5590
18–64	1020	60.0	3019	59.2	
≥65	681	40.0	2084	40.8	
Mean (SD)	59.8 (12.2)	59.7 (13.5)	0.6760		
Urbanization					0.9494
Very high	499	29.3	1467	28.8	
High	790	46.4	2411	47.3	
Moderate	286	16.8	852	16.7	
Low	126	7.4	373	7.3	
Income level (NTD^2^/per month)					0.9925
0	265	15.6	782	15.3	
1–15840	268	15.8	812	15.9	
15841–25000	779	45.8	2348	46.0	
≥25001	389	22.9	1161	22.8	
Comorbidities					
Diabetes mellitus	275	16.2	817	16.0	0.8788
Hypertension	554	32.6	1669	32.7	0.9168
Alcoholism	24	1.4	64	1.3	0.6202
Smoking-related disorder	154	9.1	454	8.9	0.8444
Chronic renal failure	33	1.9	100	2.0	0.9597
Liver cirrhosis	37	2.2	99	1.9	0.5484
Chemotherapy regimen					0.8593
Group 1 (epirubicin-based)	169	9.9	474	9.3	
Group 2 (mitomycin-based)	260	15.3	767	15.0	
Group 3 (taxanes)	20	1.2	60	1.2	
Group 4 (others)	1252	73.6	3802	74.5	
Death	849		3490		

^1^Pearson's chi-square test for categorical variables and *t*-test for continuous variables.^2^1US $ = 32.3 New Taiwan Dollars (NTD) in year 2008.

**Table 2:** Data showing overall survival in patients with gastric cancer after surgery and adjuvant chemotherapy among TCM nonusers, short-term TCM users, and long-term TCM users.

TCM use (days)	Death	Total follow-up (person-year)	Incidence rate^1^	95% CI		Mean follow-up (year)
<30 (*N* = 5103)	3490	25445.3	13715.7	13268.1	14178.4	5.0
30–179 (*N* = 1178)	630	7915.9	7958.7	7360.9	8605.1	6.7
≥180 (*N* = 523)	219	4163.1	5260.5	4608.0	6005.5	8.0

^1^Per 100,000 person-years. ^2^TCM nonusers: TCM use <30 days; short-term TCM users: TCM use 30–179 days; long-term TCM users: TCM use≥180 days.

**Table 3:** Cox model with hazard ratios and 95% confidence intervals of short-term users and long-term TCM users versus TCM nonusers.

Variables	Short-term TCM users^1^				Long-term TCM users^2^			
	HR	95% CI		*p* value	HR	95% CI		*p* value
Main model^3^	0.59	0.55	0.65	<0.0001	0.41	0.36	0.47	<0.0001
Additional covariates^4^								
Main model + diabetes mellitus	0.59	0.54	0.65	<0.0001	0.41	0.36	0.47	<0.0001
Main model + hypertension	0.59	0.54	0.64	<0.0001	0.41	0.36	0.47	<0.0001
Main model + alcoholism	0.59	0.55	0.65	<0.0001	0.41	0.36	0.47	<0.0001
Main model + smoking-related disorder	0.59	0.55	0.65	<0.0001	0.41	0.36	0.47	<0.0001
Main model + chronic renal failure	0.59	0.55	0.65	<0.0001	0.41	0.36	0.47	<0.0001
Main model + liver cirrhosis	0.59	0.55	0.65	<0.0001	0.41	0.36	0.47	<0.0001
Main model + chemotherapy regimen	0.59	0.54	0.65	<0.0001	0.41	0.36	0.47	<0.0001
Subgroup effects								
Sex								
Male	0.62	0.55	0.69	<0.0001	0.45	0.38	0.54	<0.0001
Female	0.56	0.49	0.64	<0.0001	0.36	0.29	0.45	<0.0001
Age at surgery, years								
18–64	0.60	0.54	0.67	<0.0001	0.38	0.31	0.45	<0.0001
≥65	0.58	0.51	0.66	<0.0001	0.46	0.38	0.57	<0.0001

^1^Short-term TCM users: TCM use 30–179 days. ^2^Long-term TCM users: TCM use≥180 days. ^3^Main model is adjusted for sex, age, urbanization level, and income level. ^4^Models were adjusted for covariates in the main model as well as for each additional listed covariate.

**Table 4:** Single herbs and herbal formulas most commonly prescribed for gastric cancer after surgery and adjuvant chemotherapy in our cohort.

Chinese herbal name	No. of users	%	Average duration (day)	Daily dose (g)
*Single herbs*				

Hwang Chyi (Radix Hedysari)	201	11.8	9.3	1.7
Dan-Shen (Radix Salviae Miltiorrhizae)	166	9.8	8.9	1.5
Yan-Hu-Suo (Rhizoma Corydalis)	160	9.4	7.8	1.5
Bai-Hua-She-She-Cao (Herba Hedyotidis Diffusae)	157	9.2	11.1	1.7
Hou-Pu (Cortex Magnoliae)	153	9.0	8.7	1.4

*Herbal formulas*				

Xiang-Sha-Liu-Jun-Zi-Tang	264	15.5	8.9	4.6
Ping-Wei-San	214	12.6	7.9	4.0
Ban-Xia-Xie-Xin-Tang	200	11.8	8.2	4.4
Bu-Zhong-Yi-Qi-Tang	175	10.3	9.2	4.7
Shen-Lin-Bai-Zhu-San	174	10.2	9.5	4.9

## Data Availability

The data sets used and analyzed in this study are available from the corresponding author on reasonable request.

## Abbreviations

TCM: Traditional Chinese medicine
CAM: Complementary and alternative medicine
CHPs: Chinese herbal products
NHI: National Health Insurance
NHIRD: National Health Insurance Research Database
RCIPD: Registry of Catastrophic Illness Patient Database
ICD-9-CM: International Classification of Diseases, Ninth Revision, Clinical Modification
HRs: Hazard ratios
CI: Confidence interval.
